# Up‐regulation of *DDIT4* predicts poor prognosis in acute myeloid leukaemia

**DOI:** 10.1111/jcmm.14831

**Published:** 2019-11-21

**Authors:** Zhiheng Cheng, Yifeng Dai, Yifan Pang, Yang Jiao, Yan Liu, Longzhen Cui, Liang Quan, Tingting Qian, Tiansheng Zeng, Chaozeng Si, Wenhui Huang, Jinghong Chen, Ying Pang, Xu Ye, Jinlong Shi, Lin Fu

**Affiliations:** ^1^ Department of Hematology The Second Affiliated Hospital of Guangzhou Medical University Guangzhou China; ^2^ Department of Pathology and Medical Biology University Medical Center Groningen University of Groningen Groningen Netherlands; ^3^ State Key Laboratory of Respiratory Disease Translational Medicine Center The Second Affiliated Hospital of Guangzhou Medical University Guangzhou China; ^4^ Translational Medicine Center Huaihe Hospital of Henan University Kaifeng China; ^5^ Department of Medicine William Beaumont Hospital Royal Oak MI USA; ^6^ Life Sciences Institute and Innovation Center for Cell Signaling Network Zhejiang University Hangzhou China; ^7^ Department of Biomedical Sciences University of Sassari Sassari Italy; ^8^ Department of Operations and Information Management China‐Japan Friendship Hospital Beijing China; ^9^ Department of Biomedical Engineering Chinese PLA General Hospital Beijing China; ^10^ Department of Hematology Huaihe Hospital of Henan University Kaifeng China

**Keywords:** acute myeloid leukaemia, allogeneic haematopoietic stem cell transplantation, chemotherapy, DNA damage inducible transcript 4, prognosis

## Abstract

The mammalian target of rapamycin (mTOR) inhibitor, DNA damage inducible transcript 4 (DDIT4), has inducible expression in response to various cellular stresses. In multiple malignancies, studies have shown that DDIT4 participates in tumorigenesis and impacts patient survival. We aimed to study the prognostic value of DDIT4 in acute myeloid leukaemia (AML), which is currently unclear. Firstly, The Cancer Genome Atlas was screened for AML patients with complete clinical characteristics and *DDIT4* expression data. A total of 155 patients were included and stratified according to the treatment modality and the median *DDIT4* expression levels. High *DDIT4* expressers had shorter overall survival (OS) and event‐free survival (EFS) than the low expressers among the chemotherapy‐only group (all *P* < .001); EFS and OS were similar in the high and low *DDIT4* expressers of the allogeneic haematopoietic stem cell transplantation (allo‐HSCT) group. Furthermore, in the *DDIT4* ^high^ group, patients treated with allo‐HSCT had longer EFS and OS than those who received chemotherapy alone (all *P* < .01). In the *DDIT4* ^low^ group, OS and EFS were similar in different treatment groups. Secondly, we analysed two other cytogenetically normal AML (CN‐AML) cohorts derived from the Gene Expression Omnibus database, which confirmed that high *DDIT4* expression was associated with poorer survival. Gene Ontology (GO) enrichment analysis showed that the genes related to *DDIT4* expression were mainly concentrated in the acute and chronic myeloid leukaemia signalling pathways. Collectively, our study indicates that high *DDIT4* expression may serve as a poor prognostic factor for AML, but its prognostic effects could be outweighed by allo‐HSCT.

## 1 INTRODUCTION

One of the key features of acute myeloid leukaemia (AML), a group of very aggressive myeloid malignancies, is their strikingly heterogenous outcomes.^1^ Prognostication using clinical and molecular markers is crucial in designing treatment plans. The currently used risk stratification system still has significant intragroup heterogeneity, especially in the intermediate‐risk group.^2^ Therefore, discovering appropriate biomarkers remains a research hotspot in AML. Over the years, it has been confirmed that *NPM1* and double *CEBPA* mutations are favourable biomarkers, whereas *FLT3‐ITD* mutation is associated with poor prognosis.^3^, ^4^, ^5^ In addition to genetic mutations, aberrant oncogene expressions have also been proposed as a tool for risk stratification. For example, high expressions of *sFRP2* and *DOK7* may suggest better prognosis,^6^, ^7^ but high expressions of *FHL2* and *iASPP* may indicate poor survival in AML.^8^

DNA damage inducible transcript 4 (DDIT4), also known as REDD1 or RTP801, is induced by various cellular stress conditions, such as hypoxia, endoplasmic reticulum stress, oxidative stress, heat shock and starvation.^9^ It inhibits the activity of mammalian target of rapamycin complex 1 (mTORC1), a major player in cell growth, proliferation and survival. Abnormally elevated *DDIT4* expression has been found in various malignant tumours.^10^, ^11^ Though rapamycin‐derived mTOR inhibitors are powerful drugs in treating cancer, paradoxically, the naturally occurring DDIT4 seems to protect the cancer cells from apoptosis.^10^, ^12^, ^13^ Murine lymphocytes become more sensitive to dexamethasone‐induced cell death after *DDIT4* knockdown.^12^ Additionally, *DDIT4* promotes gastric cancer proliferation and tumorigenesis through the p53 and MAPK pathways.^14^

Recent studies indicated that high *DDIT4* expression is also an adverse factor in AML.^10^, ^15^ However, the specific prognostic value of DDIT4 in AML is unknown. We aimed to evaluate the impact of *DDIT4* expression on survival and its associated gene expression patterns in AML patients treated with chemotherapy or transplantation.

## 2 MATERIALS AND METHODS

### 2.1 Patients

The first cohort included 155 de novo AML patients with *DDIT4* expression data, derived from The Cancer Genome Atlas (TCGA) database (https://cancergenome.nih.gov/).^16^ Eighty‐four patients received chemotherapy alone, whereas 71 had allogeneic haematopoietic stem cell transplantation (allo‐HSCT). The baseline clinical and molecular characteristics, follow‐up and survival data were publicly available from TCGA website, including gender, age, white blood cell (WBC) count, bone marrow (BM) and peripheral blood (PB) blast percentages, French‐American‐British (FAB) subtype, karyotype, cytogenetic risk classification, RNA and microRNA sequencing data, and gene mutation spectrum.

The second cohort contained two Gene Expression Omnibus (GEO) datasets, http://www.ncbi.nlm.nih.gov/geo/query/acc.cgi?acc=GSE6891 and http://www.ncbi.nlm.nih.gov/geo/query/acc.cgi?acc=GSE12417, including 334 and 162 patients with cytogenetically normal AML (CN‐AML), respectively. This cohort was mainly used to validate the findings of the first cohort. Affymetrix Human Genome 133 plus 2.0 and U133A gene chips were used to obtain gene expression profiles from the http://www.ncbi.nlm.nih.gov/geo/query/acc.cgi?acc=GSE6891 and http://www.ncbi.nlm.nih.gov/geo/query/acc.cgi?acc=GSE12417 datasets, and the entire process was fully compliant with the standard Affymetrix protocols. All patients’ clinical, molecular and microarray data were public accessible in Gene Expression Omnibus (://www.ncbi.nlm.nih.gov/geo).

### 2.2 Statistical analysis

Descriptive statistics were used to summarize the clinical and molecular characteristics of the patients. Datasets were described by median and/or range. Between‐group comparisons of numerical and categorical data were performed by the Mann‐Whitney *U* test and the chi‐square test, respectively. Primary endpoints were event‐free survival (EFS) and overall survival (OS). The former was defined as the time from diagnosis to the first event including relapse, death, failure to achieve complete remission, or was censored at the last follow‐up. The latter was the time from diagnosis to death from any cause, or was censored at the last follow‐up. Between‐group comparisons of OS and EFS were performed by the Kaplan‐Meier method and the log‐rank test. Multivariate Cox proportional hazard models were constructed for OS and EFS using a limited backward elimination procedure. Spearman rank correlation was used to determine the associations between gene expression profile and *DDIT4* expression. Multiple testing errors were assessed by false discovery rate (FDR). Gene Ontology (GO) enrichment analysis was conducted to assess enrichment of gene expression products associated with DDIT4. All tests were two‐tailed. Statistical significance was defined as *P* < .05. All statistical analyses were performed by R software 3.5.0, SPSS software 24.0 and GraphPad Prism software 7.0.

## 3 RESULTS

### 3.1 Differences in clinical and molecular characteristics between different *DDIT4* expression groups

In order to evaluate the prognostic significance of DDIT4 in AML, the first cohort was divided into the chemotherapy‐only group and the allo‐HSCT group. Within each group, the respective median *DDIT4* expression level was used to divide the group into high and low expression subgroups, and the clinical and molecular characteristics of subgroups were compared (Table 1).

**Table 1:** Clinical and molecular characteristics of patients in different treatment groups

Characteristics	Chemotherapy‐only group			Allo‐HSCT group		
	High DDIT4 (n = 42)	Low DDIT4 (n = 42)	*P*	High DDIT4 (n = 35)	Low DDIT4 (n = 36)	*P*
Age/years, median (range)	70 (35‐88)	63 (22‐82)	.003^a^	53 (18‐72)	50 (21‐65)	.411^a^
Age group/n (%)						
<60 y	8 (19.0)	18 (42.9)	.018^b^	23 (65.7)	29 (80.6)	.158^b^
≥60 y	34 (81.0)	24 (57.1)		12 (34.3)	7 (19.4)	
Gender/n (%)						
Male	25 (59.5)	20 (47.6)	.274^b^	20 (57.1)	21 (58.3)	.919^b^
Female	17 (40.5)	22 (52.4)		15 (42.9)	15 (41.7)	
WBC/×10^9^/L, median (range)	13.3 (1.0‐297.4)	16.1 (0.7‐171.9)	.522^a^	34.2 (0.6‐223.8)	29.4 (0.9‐115.4)	.761^a^
BM blast/%, median (range)	77 (32‐99)	66 (30‐95)	.041^a^	72 (30‐100)	70 (34‐99)	.913^a^
PB blast/%, median (range)	22 (0‐98)	25 (0‐91)	.449^a^	53 (0‐96)	45 (0‐94)	.366^a^
FAB subtypes/n (%)						
M0	7 (16.7)	0 (0.0)	.012^b^	4 (11.4)	5 (13.9)	.755^b^
M1	10 (23.8)	10 (23.8)	1.000^b^	17 (48.6)	6 (16.7)	.004^b^
M2	7 (16.7)	14 (33.3)	.078^b^	9 (25.7)	9 (25.0)	1.000^b^
M3	0 (0.0)	0 (0.0)	1.000^b^	0 (0.0)	1 (2.8)	.493^b^
M4	8 (19.0)	12 (28.6)	.306^b^	1 (2.9)	12 (33.3)	.001^b^
M5	6 (14.3)	6 (14.3)	1.000^b^	2 (5.7)	2 (5.6)	1.000^b^
M6	1 (2.4)	0 (0.0)	1.000^b^	1 (2.9)	0 (0.0)	.493^b^
M7	3 (7.1)	0 (0.0)	.241^b^	1 (2.9)	1 (2.8)	1.000^b^
Cytogenetics/n (%)						
Normal	19 (45.2)	21 (50.0)	.662^b^	18 (51.4)	15 (41.7)	.410^b^
Complex karyotype	9 (21.4)	2 (4.8)	.048^b^	9 (25.7)	2 (5.6)	.019^b^
8 Trisomy	0 (0.0)	0 (0.0)	1.000^b^	3 (8.6)	3 (8.3)	1.000^b^
inv(16)/CBFβ‐MYH11	0 (0.0)	6 (14.3)	.026^b^	0 (0.0)	5 (13.9)	.054^b^
11q23/MLL	1 (2.4)	2 (4.8)	.614^b^	1 (2.9)	2 (5.6)	1.000^b^
−7/7q‐	2 (4.8)	1 (2.4)	.614^b^	2 (5.7)	1 (2.8)	.614^b^
t(15;17)/PML‐RARA	0 (0.0)	0 (0.0)	1.000^b^	0 (0.0)	1 (2.8)	1.000^b^
t(9;22)/BCR‐ABL1	1 (2.4)	0 (0.0)	1.000^b^	1 (2.9)	1 (2.8)	1.000^b^
t(8;21)/RUNX1‐RUNX1T1	0 (0.0)	6 (14.3)	.026^b^	0 (0.0)	1 (2.8)	1.000^b^
Others	10 (23.8)	4 (9.5)	.079^b^	1 (2.9)	5 (13.9)	.199^b^
Risk/n (%)						
Good	0 (0.0)	12 (28.6)	.000^b^	0 (0.0)	7 (19.4)	.011^b^
Intermediate	26 (61.9)	20 (47.6)	.188^b^	19 (54.3)	21 (58.3)	.731^b^
Poor	16 (38.1)	10 (23.8)	.157^b^	16 (45.7)	8 (22.2)	.036^b^
*FLT3‐ITD*/n (%)						
Positive	6 (14.3)	9 (21.4)	.393^b^	9 (25.7)	8 (22.2)	.730^b^
Negative	36 (85.7)	33 (78.6)		26 (74.3)	28 (77.8)	
*NPM1*/n (%)						
Mutation	13 (31.0)	14 (13.3)	.815^b^	9 (25.7)	9 (25.0)	.945^b^
Wild type	29 (69.0)	28 (66.7)		26 (74.3)	27 (75.0)	
*DNMT3A*/n (%)						
Mutation	12 (28.6)	11 (26.2)	.807^b^	10 (28.6)	7 (19.4)	.368^b^
Wild type	30 (71.4)	31 (73.8)		25 (71.4)	29 (80.6)	
*IDH1*/*IDH2*/n (%)						
Mutation	9 (21.4)	6 (14.3)	.393^b^	11 (31.4)	6 (16.7)	.145^b^
Wild type	33 (78.6)	36 (85.7)		24 (68.6)	30 (83.3)	
*RUNX1*/n (%)						
Mutation	12 (28.6)	2 (4.8)	.003^b^	3 (8.6)	5 (13.9)	.710^b^
Wild type	30 (71.4)	42 (95.2)		32 (91.4)	31 (86.1)	
*NRAS/KRAS*/n (%)						
Mutation	6 (14.3)	6 (14.3)	1.000^b^	1 (2.9)	6 (16.7)	.107^b^
Wild type	36 (85.7)	36 (85.7)		34 (97.1)	30 (83.3)	
*TET2*/n (%)						
Mutation	4 (9.5)	7 (16.7)	.332^b^	0 (0.0)	4 (11.1)	.115^b^
Wild type	38 (90.5)	35 (83.3)		35 (100)	32 (88.9)	
*TP53*/n (%)						
Mutation	9 (21.4)	2 (4.8)	.024^b^	3 (8.6)	1 (2.8)	.357^b^
Wild type	33 (78.6)	40 (95.2)		32 (91.4)	35 (97.2)	

Abbreviations: BM, bone marrow; FAB, French‐American‐British; PB, peripheral blood; WBC, white blood cell.

a Denotes Mann‐Whitney *U* test

b Denotes chi‐square test.

In the chemotherapy‐only group, compared with the *DDIT4* ^low^ subgroup, the *DDIT4* ^high^ subgroup had more patients ≥60 years old (*P* = .018), FAB‐M0 (*P* = .012), with complex karyotype (*P* = .048), more frequent *TP53* and *RUNX1* mutations (*P* = .024, *P* = .003, respectively), and higher BM blast percentage (*P* = .041). It had fewer patients with *CBFβ‐MYH11* or *RUNX1‐RUNX1T1* (all *P* = .026) or good cytogenetic risk (*P* < .001). Gender distribution, WBC count, PB blast percentage and the frequencies of other recurrent genetic mutations (*NPM1*, *FLT3*, *NRAS/KRAS*, *IDH1/IDH2*, *DNMT3A* and *TET2*) were similar in the two subgroups.

In the allo‐HSCT group, compared with the *DDIT4* ^low^ subgroup, the *DDIT4* ^high^ subgroup had more patients with FAB‐M1 (*P* = .004), complex karyotype (*P* = .019) and poor cytogenetic risk (*P* = .036), yet fewer patients with FAB‐M4 (*P* = .001) or good cytogenetic risk (*P* = .011). Age, gender distribution, WBC count, BM/PB blast percentage and the frequencies of recurrent gene mutations (*NPM1*, *FLT3*, *RUNX1*, *DNMT3A*, *NRAS/KRAS*, *IDH1/IDH2*, *TP53* and *TET2*) were not statistically different between the two subgroups.

### 3.2 Prognostic value of *DDIT4* expression in AML

**Figure 1:**
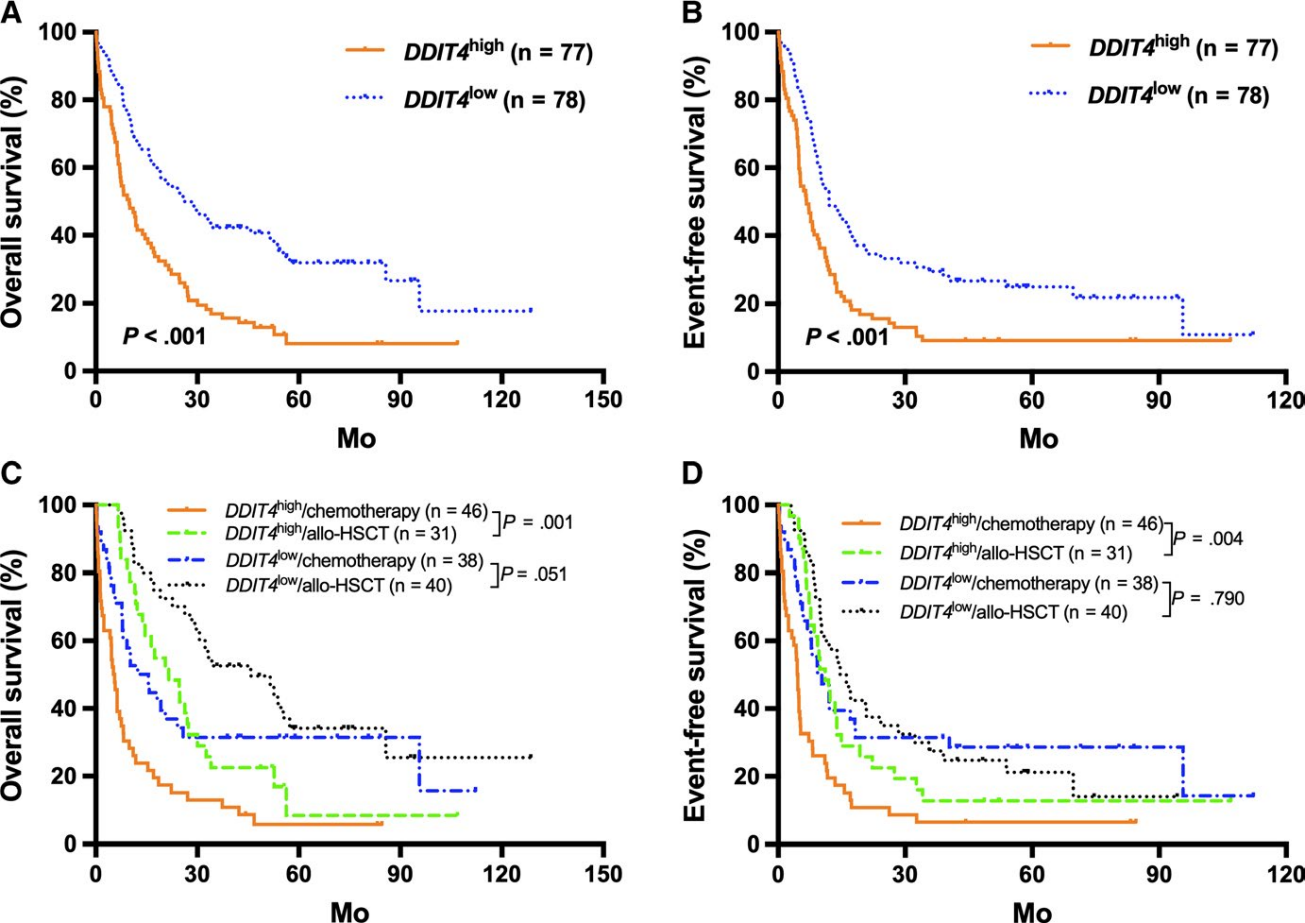
Kaplan‐Meier curves of event‐free survival (EFS) and overall survival (OS) in the first cohort. (A,B) In the entire cohort, high *DDIT4* expressers had shorter EFS and OS than the low expressers. (C,D) In the *DDIT4* ^high^ group, patients treated with allo‐HSCT had longer OS and EFS than those who received chemotherapy only. In the *DDIT4* ^low^ group, there were no significant differences in OS and EFS between different treatment groups

**Figure 2:**
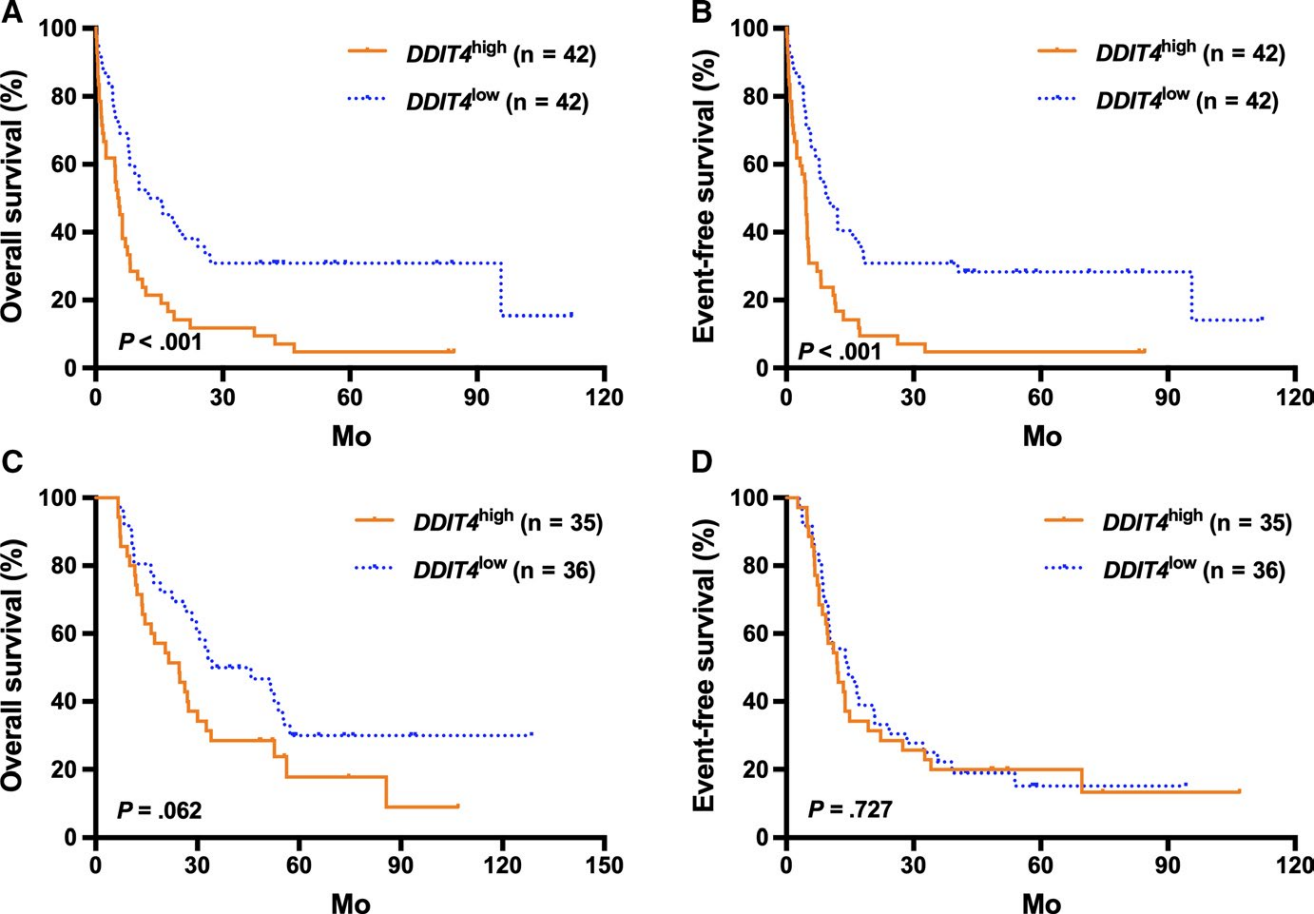
Kaplan‐Meier curves of event‐free survival (EFS) and overall survival (OS) in the chemotherapy‐only and allo‐HSCT groups. (A,B) In the chemotherapy group, high *DDIT4* expressers had shorter OS and EFS than the low expressers. (C,D) In the allo‐HSCT group, there were no significant differences in EFS and OS between high and low *DDIT4* expression groups

In the TCGA cohort, high *DDIT4* expressers generally had significantly shorter OS and EFS than the low expressers (all *P* < .001; Figure 1A,B). Then, patients were further stratified according to the treatment modality and the median *DDIT4* expression levels in the different treatment subgroups. For the high expressors (n = 77), those treated with allo‐HSCT had significantly better survival than those who received chemotherapy alone (all *P* < .01, Figure 1C,D). For the low expressors (n = 78), treatment modality did not have outstanding influence on survival (all *P* > .05, Figure 1C,D). Kaplan‐Meier analysis demonstrated that in the chemotherapy‐only group, high *DDIT4* expressers had significantly shorter OS and EFS than the low expressers (all *P* < .001, Figure 2A,B), whereas the survival was similar in the high and low expressors of the allo‐HSCT group (all *P* > .05, Figure 2C,D).

### 3.3 Multivariate analysis of possible prognostic factors

To examine whether the impact of *DDIT4* expression on AML survival was independent, we constructed multivariate Cox proportional hazard models using multiple variables, including *DDIT4* expression (high vs low), WBC count (≥15 vs <15 × 10^9^/L), age (≥60 vs <60 years), BM blasts (≥70 vs <70%), PB blasts (≥20 vs <20%), *NPM1* (mutated vs wild), *DNMT3A* (mutated vs wild), *TET2* (mutated vs wild), *TP53* (mutated vs wild) and *FLT3‐ITD* (positive vs negative) (Table 2).

**Table 2:** Multivariate analysis of EFS and OS in different treatment groups

Variables	EFS		OS	
	HR (95%CI)	*P*‐value	HR (95%CI)	*P*‐value
Chemotherapy‐only group				
*DDIT4* (high vs Low)	1.940 (1.158‐3.251)	.012	1.878 (1.118‐3.155)	.017
Age (≥60 vs <60 y)	3.051 (1.636‐5.688)	.000	2.763 (1.478‐5.167)	.001
WBC count (≥15 vs <15 × 10^9^/L)	1.470 (0.816‐2.646)	.199	1.407 (0.795‐2.490)	.241
BM blasts (≥70 vs <70%)	1.863 (1.054‐3.293)	.032	1.804 (1.025‐3.176)	.041
PB blasts (≥20 vs <20%)	0.928 (0.548‐1.571)	.781	0.868 (0.507‐1.487)	.606
*FLT3‐ITD* (positive vs negative)	1.245 (0.635‐2.442)	.523	1.183 (0.582‐2.408)	.642
*NPM1* (mutated vs wild)	0.947 (0.491‐1.826)	.871	0.772 (0.401‐1.488)	.440
*DNMT3A* (mutated vs wild)	1.704 (0.979‐2.965)	.059	1.772 (1.033‐3.042)	.038
*TET2* (mutated vs wild)	0.943 (0.429‐2.074)	.885	0.672 (0.309‐1.460)	.315
*TP53* (mutated vs wild)	3.006 (1.372‐6.587)	.006	2.444 (1.135‐5.261)	.022
Allo‐HSCT				
*DDIT4* (high vs Low)	0.995 (0.557‐1.775)	.985	1.622 (0.875‐3.004)	.124
Age (≥60 vs <60 y)	1.097 (0.546‐2.204)	.796	1.404 (0.710‐2.777)	.330
WBC count (≥15 vs <15 × 10^9^/L)	1.918 (1.032‐3.563)	.039	1.537 (0.794‐2.975)	.202
BM blasts (≥70 vs <70%)	0.777 (0.420‐1.437)	.421	0.782 (0.391‐1.566)	.488
PB blasts (≥20 vs <20%)	1.235 (0.633‐2.408)	.536	1.445 (0.695‐3.005)	.325
*FLT3‐ITD* (positive vs negative)	2.462 (1.209‐5.015)	.013	2.354 (1.048‐5.291)	.038
*NPM1* (mutated vs wild)	0.636 (0.312‐1.298)	.213	0.625 (0.277‐1.409)	.257
*DNMT3A* (mutated vs wild)	1.177 (0.605‐2.292)	.631	1.377 (0.691‐2.743)	.363
*TET2* (mutated vs wild)	0.516 (0.145‐1.838)	.307	0.961 (0.267‐3.452)	.951
*TP53* (mutated vs wild)	3.046 (0.884‐10.495)	.078	7.196 (1.871‐27.67)	.004

Abbreviations: BM, bone marrow; CI, confidence interval; EFS, Event‐free survival; HR, hazard ratio; OS, Overall survival; PB, peripheral blood; WBC, white blood cell.

In the chemotherapy‐only group, high *DDIT4* expression was an independent risk factor for both EFS and OS, along with age ≥ 60, BM blasts ≥ 70% and *TP53* mutation (all *P* < .05). In addition, *DNMT3A* mutation was an independent risk factor for OS (*P* = .038). In the allo‐HSCT group, *FLT3‐ITD* was an independent risk factor for OS and EFS (all *P* < .05), and WBC count ≥ 15 × 10^9^/L and *TP53* mutation were independent risk factors for EFS (*P* = .039) and OS (*P* = .004), respectively, but *DDIT4* expression was not an independent factor for survival.

### 3.4 Associations between genome‐wide gene expression profile and *DDIT4* expression

**Figure 3:**
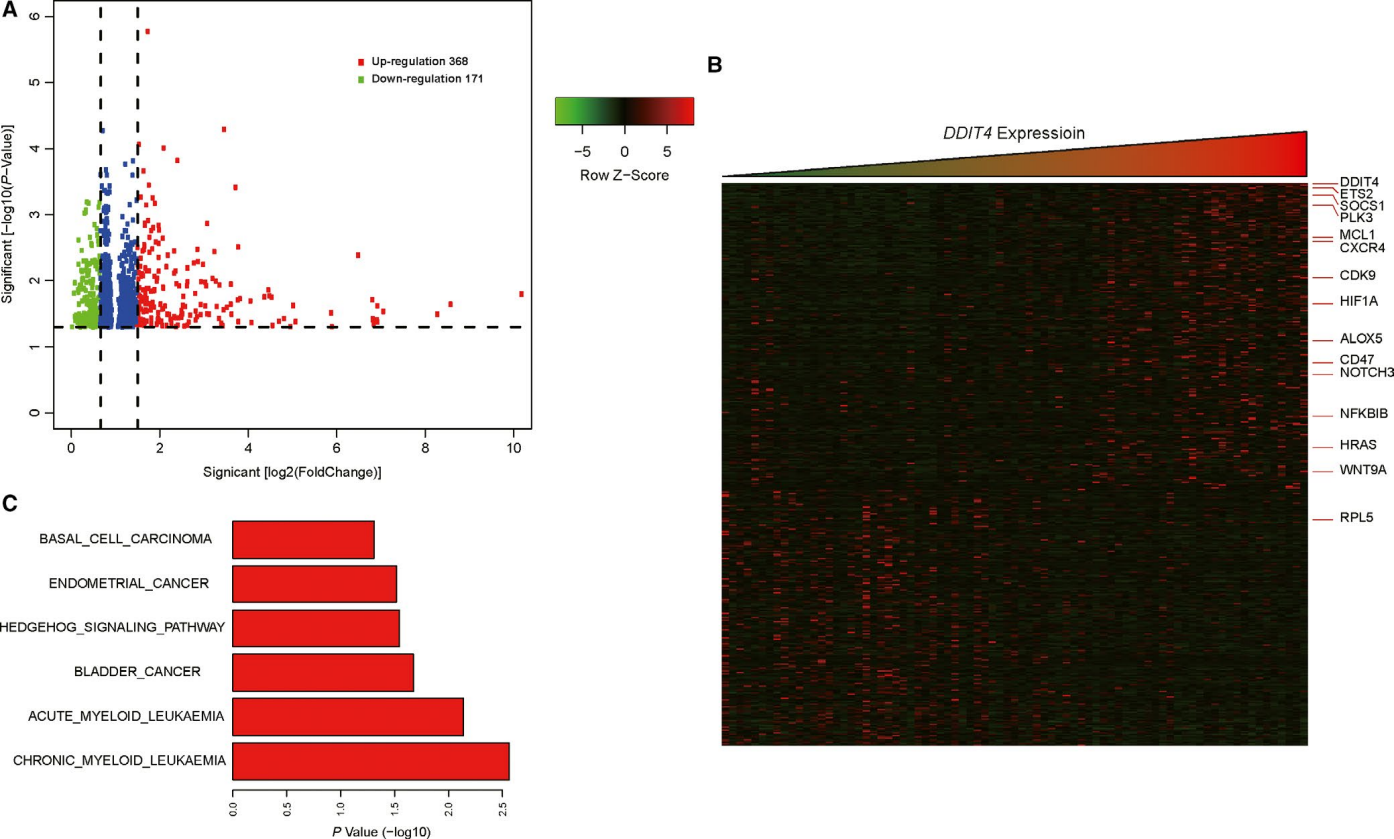
Genome‐wide gene expression profile and cell signalling pathways associated with *DDIT4* expression. (A) Volcano plot of differential gene expression. Up‐regulated and down‐regulated genes were labelled with red and green dots, respectively. (B) Heatmap of genes related to *DDIT4* expression. (C) Gene ontology (GO) enrichment analysis of genes related to *DDIT4* expression

To elucidate the possible mechanism for the influence of *DDIT4* in AML, *DDIT4*‐associated gene expression profiles were summarized utilizing the high‐throughput sequencing information from TCGA database. Three hundred and sixty‐eight up‐regulated and 171 down‐regulated genes that were significantly associated with *DDIT4* expression (*P* < .05, fold change = 1.5, Figure 3A) were screened. Eventually, with a more rigorous analysis (fold change = 2), 359 genes were excluded, and the remaining 180 genes were depicted in an aberrant expression heatmap (Figure 3B). Many leukaemia‐associated genes were positively associated with *DDIT4* expression, including *WNT9A*, *NOTCH3*, *SOCS1*, *MCL1*, *HIF1A*, *ALOX5*, *CD47*, *CXCR4*, *CDK9*, *HRAS*, *PLK3* and *ETS2*. However, *RPL5*, a tumour suppressor in multiple cancers, was negatively correlated with *DDIT4* expression. Furthermore, GO enrichment analysis suggested that the genes related to *DDIT4* expression were mainly concentrated in "acute and chronic myeloid leukaemia," "bladder cancer," "hedgehog signalling pathway," "endometrial cancer," and "basal cell carcinoma" signalling pathways (Figure 3C).

### 3.5 Validation of the prognostic value of DDIT4 expression in AML

**Figure 4:**
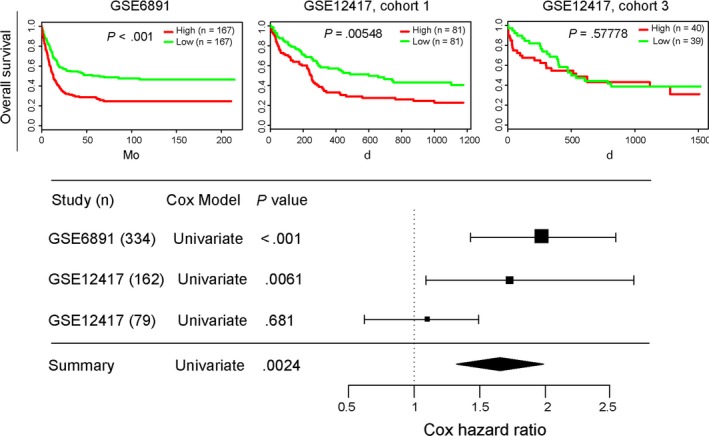
Validation of the prognostic value of *DDIT4* expression in the second cohort. High *DDIT4* expressers had shorter OS than the low expressers in two independent databases, and the combined data analysis showed the same result

In the two other large CN‐AML cohorts from the GEO database, high *DDIT4* expression was also related to significantly shorter OS. Combining the data with the TCGA cohort, results consistently showed that up‐regulated expression of *DDIT4* had deleterious effect on survival of AML patient (all *P* < .01, Figure 4).

## 4 DISCUSSION

In this retrospective study, we demonstrated that up‐regulated *DDIT4* expression adversely affects the prognosis of AML patients who underwent chemotherapy alone, but not those who were treated with allo‐HSCT, suggesting that allo‐HSCT may neutralize its negative prognostic impact. Patients with low *DDIT4* expression, on the other hand, had no survival benefit from allo‐HSCT in the study.

Dysregulated *DDIT4* expression is seen in various cancers and its role in tumorigenesis is likely tumour‐dependent, based on previous studies.^17^, ^18^, ^19^ In breast cancer, *DDIT4* is a tumour suppressor against miR‐495‐mediated oncogenesis and hypoxia resistance.^20^ In ovarian cancer, on the other hand, it is positively correlated with the oncogene p‐AKT and predicts late FIGO stage and serous adenocarcinoma,^13^ indicating its role as a tumour promotor. DDIT4 is heavily involved in the PI3K‐Akt‐mTOR signalling pathway, a crucial pathway that regulates cell growth, motility, proliferation, apoptosis and one of the most commonly altered pathways in cancer.^21^ It is a downstream effector of PI3K‐Akt‐mTOR. By collaborating with other proteins, it is responsible for prostate cancer cells’ invasive behaviour.^22^ Moreover, DDIT4 also participates in the RAS signalling pathway and highlights the complex crosstalk among different cellular signalling pathways. Overexpression of *DDIT4* after activation of RAS oncogene in RAS‐transformed human ovarian epithelial cells can promote cell proliferation and colony formation, enhance the expression of anti‐apoptotic proteins and reduce the expression of pro‐apoptotic proteins.^19^, ^23^ In our study, high *DDIT4* expression coexisted with other established poor prognostic factors, such as old age, complex karyotype and *TP53* mutation, and did not coexist with other well‐known favourable prognostic factors, such as *CBFβ‐MYH11* and *RUNX1‐RUNX1T*. As an independent adverse prognostic factor in AML patients who received chemotherapy alone, *DDIT4* up‐regulation likely plays a positive role in leukaemogenesis.

Moreover, we found that enhanced *DDIT4* expression was also an independent poor prognostic factor in CN‐AML patients with a relatively consistent cytogenetic background. GO analysis demonstrated that genes (*WNT9A*, *NOTCH3*, *SOCS1*, *MCL1*, *HIF1A*, *ALOX5*, *CD47*, *CXCR4*, *CDK9*, *HRAS*, *PLK3*, *ETS2* and *RPL5*) involved in “acute and chronic myeloid leukaemia,” “bladder cancer,” “hedgehog signalling pathway,” “endometrial cancer” and “basal cell carcinoma” signalling pathways were significantly correlated with the *DDIT4* expression. Pinto et al observed a significant positive correlation between *DDIT4* and *NOTCH1* expression, and both of them tend to highly express in high‐risk AML patients.^24^ These results indicate that *DDIT4* expression may explain some of the aggressive features of AML by involving in the above pathways, though the exact role of *DDIT4* in leukaemogenesis requires further study.

Multivariate analysis of the chemotherapy‐only group was consistent with previous studies in that older age (≥60 years), more BM blasts (≥70%), mutations in *TP53* and *DNMT3A* also independently contributed to shorter EFS and OS.^25^, ^26^, ^27^, ^28^ The effect of high *DDIT4* expression on survival was not reproduced in the allo‐HSCT group, whereas WBC count ≥ 15 × 10^9^/L, *FLT3‐ITD* and *TP53* mutation were associated with poor OS or EFS, suggesting that allo‐HSCT could ameliorate the adverse prognostic effect of high *DDIT4* expression in AML. Patients with high *DDIT4* expression benefited more from allo‐HSCT, whereas survival was not affected by treatment modality in the low expressers. Therefore, allo‐HSCT may be a better option for patients with high *DDIT4* expression, but may be not necessary for patients with low *DDIT4* expression.

To summarize, our results indicated that enhanced *DDIT4* expression could be a poor prognostic factor for AML patients treated with chemotherapy alone, and these patients might benefit from allo‐HSCT. We were able to identify a unique gene expression pattern and cell signalling pathways associated with *DDIT4* expression, which could shed lights on the role of *DDIT4* in leukaemogenesis. It is reasonable to envision it as a marker for risk stratification and guidance for treatment in AML. Our study was limited by its small, retrospective nature, and the results would need to be verified by a larger prospective population.

## CONFLICT OF INTEREST

The authors confirm that there are no conflicts of interest.

## AUTHOR CONTRIBUTIONS

Zhiheng Cheng and Yifeng Dai performed statistical analysis and drafted the manuscript. Yifan Pang and Yang Jiao critically revised the manuscript. Yan Liu, Longzhen Cui, Liang Quan, Tingting Qian, Tiansheng Zeng, Chaozeng Si, Wenhui Huang, Jinghong Chen and Ying Pang performed the acquisition and interpretation of the data. Lin Fu, Jinlong Shi and Xu Ye conceived this study and finally approved the version to be published. All authors approved the final manuscript.

## Data Availability

All data in this study were downloaded from The Cancer Genome Atlas (TCGA, https://cancergenome.nih.gov/) and Gene Expression Omnibus (GEO, ://www.ncbi.nlm.nih.gov/geo) databases. We did not involve direct interaction with patients. All analyses during this study were included in this article.

## References

[1] Liu Y , Cheng Z , Pang Y , et al. Role of microRNAs, circRNAs and long noncoding RNAs in acute myeloid leukemia. J Hematol Oncol. 2019;12:51. 31126316 10.1186/s13045-019-0734-5 PMC6534901

[2] Komanduri KV , Levine RL . Diagnosis and therapy of acute myeloid leukemia in the era of molecular risk stratification. Annu Rev Med. 2016;67:59‐72. 26473413 10.1146/annurev-med-051914-021329 PMC5701748

[3] Kunchala P , Kuravi S , Jensen R , et al. When the good go bad: Mutant NPM1 in acute myeloid leukemia. Blood Rev. 2018;32:167‐183. 29157973 10.1016/j.blre.2017.11.001

[4] Mannelli F , Ponziani V , Bencini S , et al. CEBPA‐double‐mutated acute myeloid leukemia displays a unique phenotypic profile: a reliable screening method and insight into biological features. Haematologica. 2017;102:529‐540. 28250006 10.3324/haematol.2016.151910 PMC5394975

[5] Döhner H , Estey E , Grimwade D , et al. Diagnosis and management of AML in adults: 2017 ELN recommendations from an international expert panel. Blood. 2017;129:424‐447. 27895058 10.1182/blood-2016-08-733196 PMC5291965

[6] Aly RM , Taalab MM , Abdsalam EM . Prognostic significance of secreted frizzled‐related protein 2 expression in cytogenetically normal primary acute myeloid leukemia. Am J Med Sci. 2015;350:369‐373. 26517501 10.1097/MAJ.0000000000000567

[7] Zhang L , Li R , Hu K , et al. Prognostic role of DOK family adapters in acute myeloid leukemia. Cancer Gene Ther. 2019;26(9‐10):305‐312. 30348947 10.1038/s41417-018-0052-z

[8] Cheng Z , Dai Y , Pang Y , et al. Enhanced expressions of FHL2 and iASPP predict poor prognosis in acute myeloid leukemia. Cancer Gene Ther. 2019;26:17‐25. 29910468 10.1038/s41417-018-0027-0

[9] Tirado‐Hurtado I , Fajardo W , Pinto JA . DNA damage inducible transcript 4 gene: the switch of the metabolism as potential target in cancer. Front Oncol. 2018;8:106. 29707520 10.3389/fonc.2018.00106 PMC5906527

[10] Pinto JA , Rolfo C , Raez LE , et al. In silico evaluation of DNA Damage Inducible Transcript 4 gene (DDIT4) as prognostic biomarker in several malignancies. Sci Rep. 2017;7:1526. 28484222 10.1038/s41598-017-01207-3 PMC5431475

[11] Barakat DJ , Mendonca J , Barberi T , et al. C/EBPβ regulates sensitivity to bortezomib in prostate cancer cells by inducing REDD1 and autophagosome‐lysosome fusion. Cancer Lett. 2016;375:152‐161. 26968249 10.1016/j.canlet.2016.03.005 PMC4818955

[12] Molitoris JK , McColl KS , Swerdlow S , et al. Glucocorticoid elevation of dexamethasone‐induced gene 2 (Dig2/RTP801/REDD1) protein mediates autophagy in lymphocytes. J Biol Chem. 2011;286:30181‐30189. 21733849 10.1074/jbc.M111.245423 PMC3191057

[13] Jia W , Chang B , Sun L , et al. REDD1 and p‐AKT over‐expression may predict poor prognosis in ovarian cancer. Int J Clin Exp Pathol. 2014;7:5940‐5949. 25337238 PMC4203209

[14] Du F , Sun L , Chu Y , et al. DDIT4 promotes gastric cancer proliferation and tumorigenesis through the p53 and MAPK pathways. Cancer Commun (Lond). 2018;38:45. 29976242 10.1186/s40880-018-0315-y PMC6034313

[15] Zhao X , Li Y , Wu H . A novel scoring system for acute myeloid leukemia risk assessment based on the expression levels of six genes. Int J Mol Med. 2018;42:1495‐1507. 29956722 10.3892/ijmm.2018.3739 PMC6089755

[16] Cancer Genome Atlas Research Network , Ley TJ , Miller C , et al. Genomic and epigenomic landscapes of adult de novo acute myeloid leukemia. N Engl J Med. 2013;368:2059‐2074. 23634996 10.1056/NEJMoa1301689 PMC3767041

[17] Zoncu R , Efeyan A , Sabatini DM . mTOR: from growth signal integration to cancer, diabetes and ageing. Nat Rev Mol Cell Biol. 2011;12:21‐35. 21157483 10.1038/nrm3025 PMC3390257

[18] Hu YY , Liu JC , Xing AY , et al. REDD1 expression in placenta during human gestation. Reprod Sci. 2012;19:995‐1000. 22527987 10.1177/1933719112440054

[19] Chang B , Liu G , Yang G , et al. REDD1 is required for RAS‐mediated transformation of human ovarian epithelial cells. Cell Cycle. 2009;8:780‐786. 19221489 10.4161/cc.8.5.7887

[20] Hwang‐Verslues WW , Chang PH , Wei PC , et al. miR‐495 is upregulated by E12/E47 in breast cancer stem cells, and promotes oncogenesis and hypoxia resistance via downregulation of E‐cadherin and REDD1. Oncogene. 2011;30:2463‐2474. 21258409 10.1038/onc.2010.618

[21] Porta C , Paglino C , Mosca A . Targeting PI3K/Akt/mTOR signaling in cancer. Front Oncol. 2014;4:64. 24782981 10.3389/fonc.2014.00064 PMC3995050

[22] Schwarzer R , Tondera D , Arnold W , et al. REDD1 integrates hypoxia‐mediated survival signaling downstream of phosphatidylinositol 3‐kinase. Oncogene. 2005;24:1138‐1149. 15592522 10.1038/sj.onc.1208236

[23] Smith ER , Xu XX . REDD1, a new Ras oncogenic effector. Cell Cycle. 2009;8:675‐676. 10.4161/cc.8.5.8184 19242117

[24] Pinto JA , Bravo L , Chirinos LA , et al. Expression of DDIT4 is correlated with NOTCH1 and high molecular risk in acute myeloid leukemias. Blood. 2016;128:5254.

[25] Cheng Z , Hu K , Tian L , et al. Clinical and biological implications of mutational spectrum in acute myeloid leukemia of FAB subtypes M4 and M5. Cancer Gene Ther. 2018;25:77‐83. 29491461 10.1038/s41417-018-0013-6

[26] Bacher U , Haferlach C , Alpermann T , et al. Comparison of genetic and clinical aspects in patients with acute myeloid leukemia and myelodysplastic syndromes all with more than 50% of bone marrow erythropoietic cells. Haematologica. 2011;96:1284‐1292. 21606170 10.3324/haematol.2011.043687 PMC3166098

[27] Marcucci G , Metzeler KH , Schwind S , et al. Age‐related prognostic impact of different types of DNMT3A mutations in adults with primary cytogenetically normal acute myeloid leukemia. J Clin Oncol. 2012;30:742‐750. 22291079 10.1200/JCO.2011.39.2092 PMC3295550

[28] Kadia TM , Jain P , Ravandi F , et al. TP53 mutations in newly diagnosed acute myeloid leukemia: Clinicomolecular characteristics, response to therapy, and outcomes. Cancer. 2016;122:3484‐3491. 27463065 10.1002/cncr.30203 PMC5269552

